# The Endothelin Type A Receptor as a Potential Therapeutic Target in Preeclampsia

**DOI:** 10.3390/ijms18030522

**Published:** 2017-02-28

**Authors:** Bhavisha Bakrania, Jeremy Duncan, Junie P. Warrington, Joey P. Granger

**Affiliations:** Department of Physiology and Biophysics, University of Mississippi Medical Center, Jackson, MS 39216, USA; bbakrania@umc.edu (B.B.); jduncan2@umc.edu (J.D.); jpwarrington@umc.edu (J.P.W.)

**Keywords:** preeclampsia, pregnancy, hypertension, endothelin, endothelium, placenta, cardiovascular, blood pressure, vascular smooth muscle

## Abstract

Preeclampsia (PE) is a disorder of pregnancy typically characterized by new onset hypertension after gestational week 20 and proteinuria. Although PE is one of the leading causes of maternal and perinatal morbidity and death worldwide, the mechanisms of the pathogenesis of the disease remain unclear and treatment options are limited. However, there is increasing evidence to suggest that endothelin-1 (ET-1) plays a critical role in the pathophysiology of PE. Multiple studies report that ET-1 is increased in PE and some studies report a positive correlation between ET-1 and the severity of symptoms. A number of experimental models of PE are also associated with elevated tissue levels of prepro ET-1 mRNA. Moreover, experimental models of PE (placental ischemia, sFlt-1 infusion, Tumor necrosis factor (TNF) -α infusion, and Angiotensin II type 1 receptor autoantibody (AT1-AA) infusion) have proven to be susceptible to Endothelin Type A (ET_A_) receptor antagonism. While the results are promising, further work is needed to determine whether ET antagonists could provide an effective therapy for the management of preeclampsia.

## 1. Introduction

Preeclampsia (PE) is a pregnancy-specific disorder characterized by new onset hypertension after gestational week 20 and proteinuria [1]. However, in the absence of proteinuria, PE is also diagnosed as hypertension associated with thrombocytopenia, impaired liver function, development of renal insufficiency, pulmonary edema, or new onset cerebral or visual disturbances [1]. Despite being a leading contributor of maternal and perinatal morbidity and death worldwide, the mechanisms of the pathogenesis of preeclampsia remain unclear and treatment options are limited [1,2,3]. Current therapy for the management of preeclampsia includes antihypertensives such as methyldopa, labetalol, and nifedipine and magnesium sulfate for prevention of eclamptic seizures [1]; however, these treatments have limited efficacy, and the only “cure” for preeclampsia is the delivery of the placenta. Early onset PE (<34 weeks) is more severe, and outcomes are especially dire if disease develops very preterm (<32 weeks). Each week pregnancy is prolonged markedly reduces fetal morbidity and mortality, but only at the expense of an increased risk of maternal demise. A therapeutic approach that will benefit, yet do no harm to both mother and fetus, represents a critically important unmet medical need.

## 2. The Pathogenesis of Preeclampsia

While the root causes of preeclampsia remain unknown, it is thought that in some cases of the disease, especially early onset PE, abnormal placentation due to insufficient trophoblast invasion and failure of spiral artery remodeling leads to inadequate blood flow to the placenta and a continuing cycle of repeated ischemia-reperfusion injury [2,3,4]. The resulting hypoxic environment within the placenta stimulates oxidative stress and the release of placental factors such as soluble fms-like tyrosine kinase 1 (sFlt-1), soluble endoglin, agonistic autoantibodies to the angiotensin type 1 receptor (AT1-AA), and inflammatory cytokines [2,3,4,5,6,7,8]. These factors, along with the presence of additional maternal risk factors for preeclampsia, such as age, obesity, and pre-existing hypertension contribute to a generalized systemic vascular endothelial dysfunction and result in increased systemic vascular resistance and hypertension.

## 3. The Endothelin System in Preeclampsia

Increasing evidence suggests that endothelin-1 plays an important role (ET-1) in the pathophysiology of preeclampsia [9,10,11]. ET-1 was identified as the most potent vasoconstrictor known [12]. ET-1 elicits its actions through two cell-surface G-protein-coupled receptors, ET-1 Type A (ET_A_), located primarily on vascular smooth muscle cells, and Type B (ET_B_) receptors located on endothelial, vascular smooth muscle, and renal epithelial cells. Signaling through the ET_A_ receptor results in cell proliferation and vasoconstriction, while activation of the ET_B_ receptor mediates vasodilation and natriuresis via nitric oxide and prostacyclin [12].

Multiple studies have examined circulating ET-1 levels in normal pregnant and preeclamptic women, and found elevated plasma ET-1 in the preeclamptic group, with some studies indicating that circulating ET-1 correlates with the severity of the disease symptoms, though this is not a universal finding [9,10,11,13,14,15]. Verdonk et al. recently investigated the relationship between disturbed angiogenic balance, arterial pressure, and ET-1 in pregnant women with a high (≥85; *n* = 38) or low (<85) soluble Fms-like tyrosine kinase-1/placental growth factor ratio [16]. Plasma ET-1 levels were increased in women with a high ratio. In addition, plasma ET-1 correlated positively with soluble fms-like tyrosine kinase-1. Aggarwal et al. also investigated the correlation between ET-1 and sFlt-1, placental growth factor (PlGF), and soluble endoglin (sEng) levels during uncomplicated normotensive pregnancy and PE [17]. Their results also show an association between elevation of sFlt-1, sEng, and ET-1 in the maternal circulation in PE, which strengthens the possibility that ET-1 could be a mediator in pathogenesis of PE syndrome secondary to the anti-angiogenic factors, sFlt-1 and sEng, released by the placenta [17].

A number of experimental models of preeclampsia are also associated with elevated tissue levels of prepro ET-1 mRNA. Both the renal cortex and medulla of placental ischemic rats express significantly higher levels of the ET-1 precursor, preproendothelin, when compared to normal pregnant controls [18]. Furthermore, chronic elevation of sFlt-1 in pregnant rats directly increased preproendothelin gene expression in the renal cortex [8]. It has also been shown that infusion of tumor necrosis factor-α (TNF-α) directly induced hypertension in pregnant rats and is associated with significant increases in the expression of preproendothelin in the maternal vasculature, placenta, and kidney [7]. Finally, direct infusion of the AT1-AA into pregnant rats results in moderate hypertension, and is associated with increased preproendothelin expression in both the renal cortex and placenta [19].

Matrix metalloproteinases (MMPs) are enzymes that cleave the ET-1 precursor, big ET-1 (bET-1) to active ET-1, and could therefore be another potential mechanism for increased ET-1 levels in PE. Interestingly, the expression levels of MMPs (particularly MMP-2 and MMP-1), have been shown to increase in women who subsequently develop PE. Abdalvand et al. hypothesized that the increased MMP-2 expression leads to increased bET-1 conversion, and therefore increasing vasoconstriction [20]. They reported increased vascular contractility to bET-1 in the reduced uterine perfusion pressure (RUPP) model of PE, likely driven by upstream enzymatic activity. Furthermore, the greater contribution of MMP to cleave bET-1 to ET-1 ex vivo in RUPP suggests that this enzyme could contribute to bET-1–induced contractility. Nugent et al. also recently examined the potential role of MMP-1 as an activator of protease-activated receptor 1 (PAR-1), which is known to mediate the release of ET-1 in endothelial cells [21]. They reported increased serum and vascular MMP-1 in women with PE and hypothesized that the action of MMP-1 on PAR-1 might have vasoconstrictive effects. They demonstrated that MMP-1 increases vascular reactivity in response to vasoconstrictor hormones, via an endothelial ET-1 pathway. The authors concluded that increased levels of MMP-1 in the circulation and expression in blood vessels may contribute to the pathogenesis of hypertension in preeclamptic women.

## 4. ET_A_ Receptor Antagonism in Animal Models Used to Study the Pathophysiology of PE

A number of experimental animal models have been utilized to examine the etiology and development of preeclampsia [22,23,24]. One which we and others have used with great success is the reduced uterine perfusion pressure (RUPP) model, where mechanical restriction of blood flow to the placenta, causes hypoxia and ischemia [24]. This model that mimics numerous pathophysiological characteristics of PE, has been used in species ranging from rats to non-human primates. These models display hypertension, angiogenic imbalance, renal injury, proteinuria, and endothelial dysfunction [8,18,25,26]. The renal cortex and medulla of RUPP rats express significantly higher ET-1 precursor, preproendothelin mRNA, when compared to normal pregnant controls [18]. We have also reported that serum from RUPP animals enhanced ET-1 production from endothelial cells. These data suggest that circulating factors produced by placental ischemia are responsible for increased vascular ET-1 production [27]. Administration of ET_A_ receptor antagonist abolished hypertension in RUPP rats (Table 1), and there was a trend for improved renal function [18]. We have also reported that RUPP sera significantly induced production of ET-1 from the endothelial cells when compared to those exposed to serum from normal pregnant animals, suggesting that circulating factors produced by placental ischemia are responsible for increased vascular ET-1 production [27]. When an ET_A_ receptor antagonist was administered to the RUPP rats, the associated hypertension was abolished (Table 1), and there was a trend for improved renal function [18]. Tam Tam et al. also reported that pretreatment with ET_A_ attenuated both the mean arterial pressure (MAP) and uterine artery resistance index (UARI) in the RUPP group without affecting these parameters in the normal pregnant (NP) group [28]. The improvement in UARI could be one potential mechanism for the reduction in MAP in response to ET_A_ in pregnant dams with ischemic placentas. Collectively, these studies suggest that placental ischemia induces factors that activate ET-1 production in vessels, via ET_A_ receptors, contributing to maternal hypertension seen in the RUPP model. Zhou et al. also demonstrated that chronic hypoxia during gestation triggers preeclampsia-like symptoms in pregnant rats via heightened ET-1- and ET_A_ receptor-mediated signaling, providing a molecular mechanism linking gestational hypoxia and increased risk of preeclampsia [29].

The role of the ET-1 system has been studied in models of sFlt-1 overexpression in pregnancy. Our group has recently reported that continuous infusion of sFlt-1 in pregnant rats directly increased the level of ET-1 in the renal cortex and caused an increase in the MAP of ~20 mmHg [8]. Co-administration of an ET_A_ receptor antagonist with sFlt-1 completely abolished hypertension (Table 1), strongly supporting ET-1 as an important mediator of sFlt-1 induced hypertension [8]. Kappers et al. also reported that the administration of Sunitinib, a tyrosine kinase inhibitor of the vascular endothelial growth factor (VEGF) receptor, induces a reversible rise in blood pressure (BP) in patients, and also in rats, associated with activation of the endothelin-1 system and generalized microvascular dysfunction [30]. Moreover, VEGF inhibition with Sunitinib in swine results in endothelin-mediated hypertension [31]. Thus, another potential mechanism whereby VEGF blockade could increase BP is by enhancing ET-1 synthesis.

Increased production of TNF-α, a component of the innate immune response, is seen in both women and rodents undergoing chronic placental ischemia [7,32,33]. Prior studies in vitro demonstrated that production of ET-1 by endothelial cells could be mediated by exposure to TNF-α [27]. Studies from our group have demonstrated that administration of the soluble TNF-α receptor, Etanercept, reduces hypertension associated with placental ischemia in pregnant rats [7]. This treatment is accompanied by reduced expression of the preproendothelin in the renal cortex and medulla as well as the placenta itself [7]. It has also been shown that infusion of TNF-α alone induces hypertension in pregnant rats, producing an approximate 20 mmHg increase in MAP in late gestation [34]. This is also associated with a significant increase in the expression of preproendothelin in the maternal vasculature, placenta, and kidney. As seen in the RUPP model, co-administration of an ET_A_ receptor antagonist in these animals completely abolished the associated hypertension [7]. Wang et al. also reported that LIGHT, a novel tumor necrosis factor superfamily member, is significantly elevated in the circulation and placentas of preeclamptic women [35]. Injection of LIGHT into pregnant mice causes placental apoptosis, small fetuses, and key features of preeclampsia, hypertension and proteinuria. The work reported in this study was the first to show that elevated LIGHT, coupled with enhanced lymphotoxin β and herpes virus entry mediator receptor activation, promotes placental damage and triggers the release of potent vasoactive factors (sFlt-1 and ET-1) [35]. Together, these data suggest that TNF-induced hypertension is mediated by ET-1, through ET_A_ receptor activation.

Studies show that infusion of the AT1-AA directly into pregnant rats also causes moderate hypertension and is accompanied by elevated preproendothelin in the placenta and renal cortex. AT1-AA-induced hypertension is abolished by ET_A_ receptor antagonist administration (Table 1), highlighting the importance of the ET-1 system in this model [19]. Zhou et al. found that IgG from preeclamptic women induced preproET-1 mRNA expression that was blocked by angiotensin II type 1 receptor antagonists in pregnant mice [36]. The ET_A_ receptor-specific antagonist BQ123 significantly reduced autoantibody-induced hypertension, proteinuria, and renal damage, suggesting that ET-1 plays an important role in the pathophysiology of PE.

Mazzuca et al. recently reported that endothelial ET_B_ receptor expression/activity is reduced in pregnant rats with RUPP and suggested that these findings may explain the increased BP and ET-1 vasoconstriction and reduced ET_B_ receptor-mediated relaxation in placental ischemia-induced hypertension [37]. The authors proposed that the ET_B_ receptor could be an important target in preeclampsia and upregulation of endothelial ET_B_ receptor, using pharmacological agonists or genetic manipulation, may represent a novel approach in managing preeclampsia.

## 5. The ET_A_ Receptor as a Potential Therapeutic Target in Preeclampsia?

ET receptor antagonists are used in the treatment of numerous cardiovascular diseases including pulmonary and systemic hypertension, congestive heart failure, myocardial infarction, vascular restenosis and atherosclerosis, renal failure, cerebrovascular disease and cancer. Given the myriad of experimental models of PE that are ameliorated by ET_A_ antagonism, could the ET-1 system be a therapeutic target for the management of hypertension in PE? Excitement at this approach has been tempered by work showing that genetic knockout of the ET_A_ receptor leads to birth defects and eventual embryonic lethality in mice [38]. As a result, administration of endothelin receptor antagonists is contraindicated in pregnancy [39]. However, specific windows during early to mid-gestation have been identified in animal studies, where pharmacological antagonism of ET_A_ caused phenotypes similar to that seen in the knockout. Administration of the ET_A_ antagonist only during late gestation was not performed, and it is entirely plausible that ET_A_ receptor antagonists might prove safe and efficacious in later pregnancy, when the symptoms of preeclampsia are most severe [40]. Furthermore, development of ET_A_ receptor antagonists that do not traverse the feto–placental barrier would avoid these complications altogether. Indeed, Thaete and colleagues recently reported an endothelin receptor antagonist that had limited access to the fetal compartment during chronic maternal administration late in pregnancy [41].

## 6. Conclusions

**Figure 1:**
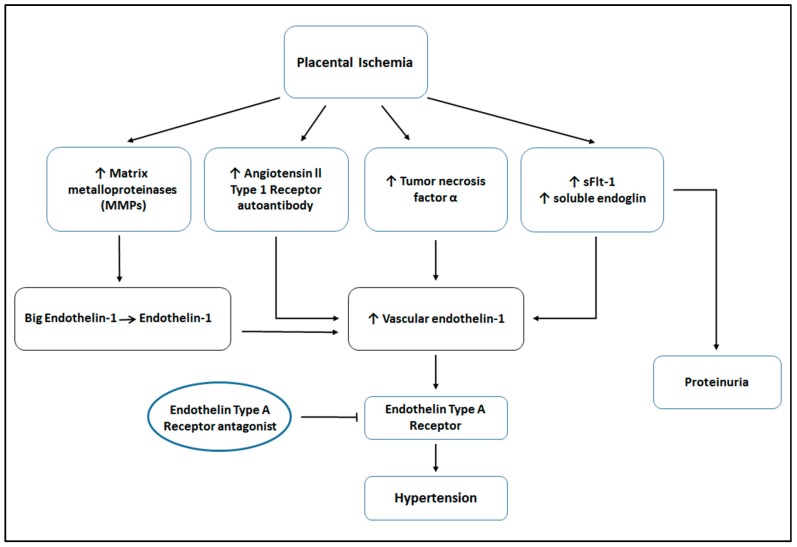
Factors leading to hypertension in response to placental ischemia. Placental ischemia is associated with increases in several factors, such as matrix metalloproteinases (MMPs), angiotensin II type 1 receptor autoantibody (AT1-AA), tumor necrosis factor-α (TNF-α), soluble endoglin, and soluble fms-like tyrosine kinase-1 (sFlt-1). MMPs cleave big endothelin to active endothelin. These circulating factors lead to increased levels of vascular endothelin-1 (ET-1) expression and subsequent ET_A_ activation, resulting in hypertension. ET antagonists may reduce hypertension, therefore improving maternal and fetal outcomes.

In summary, despite being one of the leading causes of maternal and perinatal morbidity and death worldwide, the mechanisms of the pathogenesis of preeclampsia remain unclear and treatment options are limited. Increasing evidence suggests an important role for endothelin-1 (ET-1) in the pathophysiology of preeclampsia. Several studies show elevated ET-1 in the plasma of preeclamptic women, with some studies indicating that circulating levels of ET-1 correlates with the severity of the disease symptoms. Interestingly, a number of placental factors associated with placental ischemia and preeclampsia are also correlated with elevated tissue levels of ET-1 mRNA (see Figure 1). More importantly, experimental models of preeclampsia have proven to be susceptible to ET_A_ receptor antagonism. While the results are promising, further work is needed to determine whether ET antagonism could offer an effective therapeutic target for the treatment of preeclampsia.

## Figures and Tables

**Table 1:** Summary of responses to ET_A_ receptor antagonists in animal models of preeclampsia.

Animal Model	Response to ET_A_ Receptor Antagonist	References
RUPP	↓ MAP, ↓ UARI	[18,28]
sFlt-1 infusion	↓ MAP	[8]
TNF-α infusion	↓ MAP	[7]
AT1-AA infusion	↓ MAP	[19]

RUPP, reduced uterine perfusion pressure; sFlt-1, soluble fms-like tyrosine kinase-1; TNF-α, tumor necrosis factor-α; AT1-AA, angiotensin II receptor type 1 autoantibody; ETA, endothelin receptor type A; MAP, mean arterial pressure; UARI, uterine artery resistance index; ↓, reduced.

## Abbreviations

PE	Preeclampsia
sFlt-1	Soluble fms-like tyrosine kinase 1
TNF-α	Tumor necrosis factor-α
AT1-AA	Angiotensin II type 1 receptor autoantibody
ET-1	Endothelin-1
PlGF	Placental growth factor
sEng	Soluble Endoglin
bET-1	Big Endothelin-1
MMP	Matrix Metalloproteinase
RUPP	Reduced Uterine Perfusion Pressure
ET_A_	Endothelin Type A receptor
ET_B_	Endothelin Type B receptor
PAR-1	Protease-activated receptor 1
MAP	Mean Arterial Pressure
UARI	Uterine Artery Resistance Index
NP	Normal Pregnant
BP	Blood Pressure
VEGF	Vascular Endothelial Growth Factor
